# Relationship of telomere length in colorectal cancer patients with cancer phenotype and patient prognosis

**DOI:** 10.1038/s41416-019-0525-3

**Published:** 2019-07-17

**Authors:** Michal Kroupa, Sivarama Krishna Rachakonda, Vaclav Liska, Nalini Srinivas, Marketa Urbanova, Katerina Jiraskova, Michaela Schneiderova, Ondrej Vycital, Veronika Vymetalkova, Ludmila Vodickova, Rajiv Kumar, Pavel Vodicka

**Affiliations:** 1 Department of Molecular Biology of Cancer, Institute of Experimental Medicine, The Czech Academy of Sciences, Prague, Czech Republic; 2 Department of Histology and Embryology, Faculty of Medicine in Pilsen, Charles University, Pilsen, Czech Republic; 3 Division of Molecular Genetic Epidemiology, German Cancer Research Center, Heidelberg, Germany; 4 Faculty of Medicine and Biomedical Center in Pilsen, Charles University, Pilsen, Czech Republic; 5 Institute of Biology and Medical Genetics, First Faculty of Medicine, Charles University, Prague, Czech Republic; 6 Department of Surgery, General University Hospital in Prague, Prague, Czech Republic

**Keywords:** Colorectal cancer, Colorectal cancer

## Abstract

**Background:**

Telomeres, repetitive DNA capping ends of eukaryotic chromosomes, are important in the maintenance of genomic integrity. Perturbed telomeres are common features of many human malignancies, including colorectal cancer.

**Methods:**

Telomere length (TL), measured by a Monochrome Multiplex Real-Time qPCR, was investigated in tumour tissues, adjacent mucosa, and blood from patients with colorectal cancer with different clinicopathological features and its impact on patient survival. TL was also measured in a limited number of liver metastases, non-cancerous liver tissues or corresponding tissues from the same patients.

**Results:**

TL in tumour tissues was shorter than in the adjacent mucosa (*P* < 0.0001). Shorter TL was observed in tumours with lower stage than in those with advanced stages (*P* = 0.001). TL was shorter in tumours at the proximal than at the distal sites of the colon (*P* < 0.0001). Shorter TL was also associated with microsatellite instability (*P* = 0.001) and mucinous tumour histology (*P* < 0.0001). Patients with a smaller TL ratio between tumour tissues and the adjacent mucosa were associated with increased overall survival (*P* = 0.022). Metastasised tumours had shorter telomeres than the adjacent non-cancerous liver tissues (*P* = 0.0005).

**Conclusions:**

Overall, the results demonstrate differences in TL between tumours and the adjacent mucosa, between tumours located at different sites and association with patient survival.

## Background

Telomeres, tandem G-rich hexanucleotide repeats that are involved in the maintenance of genome integrity, undergo a progressive shortening through successive cell division. Gradual telomeric attrition is caused by incomplete DNA replication of a lagging strand. Telomere length (TL) is also affected by the genotoxic effect of environmental and intracellular DNA-damaging agents, including anticancer drugs.^1–3^ Telomere shortening correlates with age.^4^ Tumour cells due to increased proliferation undergo faster telomeric attrition than non-cancerous somatic cells. Telomere shortening can act as a potent tumour-suppressing mechanism, limiting cells from uncontrolled growth. However, most cancers evolve a mechanism to overcome the proliferative barrier, due to telomere attrition through telomerase rejuvenation. The rejuvenated telomerase preferentially stabilises the shortest telomeres and critically short telomeres can lead to the formation of anaphase bridges through breakage–fusion–bridge cycles that contributes to chromosome instability (CIN).^5^

Comprehensive reviews clearly documented that colorectal cancer (CRC) may not be considered as a homogeneous disease.^6,7^ CIN along with microsatellite instability (MSI) represents two major pathways prevalent in the genesis of CRC,^6^ as well as patterns of epigenetic alterations constitute CRC heterogeneity. Particular molecular features of CRC, attributable to the specific segments of the bowel, may affect TL in the target tissue. Impaired mismatch repair (MMR) leading to MSI is characterised by excessive indel mutations in microsatellite sequences,^8^ particularly in the proximal colon. It is possible that the compromised MMR pathway could affect telomeric repeats as well.^9^ Due to the importance of telomere biology in cancer initiation, progression and patient prognosis, inconsistency of the current results in this field^10,11^ and the advent of therapeutical concepts based on targeting telomere or telomerase inhibition in order to overcome resistance, lack of drug sensitivity and toxicity, it is imperative to understand the status of TL and the factors affecting it in tumour tissue of CRC patients.

In this study, we determined TL in tumours and the adjacent mucosa in order to find a correlation, if any. We also investigated the impact of tumour location (reflecting CRC heterogeneity) on TL. The impact of TL in tumour/mucosa on the overall patient survival was also determined.

## Methods

### Population characteristics

Sporadic CRC patients (*N* = 721) with histologically confirmed tumours were included in the study. Personal data that included date of birth, sex, and diabetes mellitus were obtained using a structured questionnaire. For all patients, clinical data including tumour-related parameters, such as the tumour location, International Union against Cancer (UICC) TNM stage system status, degree of tumour differentiation, were collected along with information about distant metastases, relapse, and date of death.

Patients were recruited from 2004 to 2014 in different oncological and gastroenterological departments of various hospitals within the Czech Republic. The last update of patient follow-up for this study was December 2015. The description of the studied population including fundamental characteristics of CRC and background variables is presented in Table 1.

**Table 1:** RTL values and clinicopathological characteristics of the studied population

		RTL ratio (RTL tumour/RTL adjacent mucosa)		Age adjusted		RTL tumour		Age adjusted		RTL adjacent mucosa		Age adjusted
		Mean ± SD	Median [IQR]	*P* value		Mean ± SD	Median [IQR]	*P* value		Mean ± SD	Median [IQR]	*P* value
	All				All				All			
Sex	*n* = 661^a^				*n* = 696				*n* = 677			
Male	415	0.85 ± 0.56	0.75 [0.51–1.03]		438	1.27 ± 0.92	1.01 [0.69–1.54]		423	1.94 ± 1.73	1.30 [0.83–2.26]	
Female	246	0.76 ± 0.40	0.72 [0.48–0.98]	0.036^a^	258	1:21 ± 0.91	0.99 [0.64–1.40]	0.38	254	1.94 ± 1.57	1.32 [0.86–2.42]	0.98
Smoking status	*n* = 635				*n* = 671				*n* = 650			
Smokers	276	0.89 ± 0.58	0.78 [0.54–1.08]		293	1.29 ± 1.01	1.01 [0.70–1.49]		285	1.81 ± 1.51	1.25 [0.85–2.16]	
Nonsmokers	359	0.76 ± 0.44	0.70 [0.46–0.95]	0.0051^a^	378	1.25 ± 0.86	1.02 [0.66–1.56]	0.99	365	2.09 ± 1.80	1.42 [0.87–2.58]	0.017^a^
Tumour stage	*n* = 634				*n* = 670				*n* = 650			
I (Ref)	88	0.67 ± 0.34	0.62 [0.39–0.90]		95	1.08 ± 0.78	0.86 [0.58–1.39]		91	2.10 ± 2.00	1.35 [0.76–2.10]	
II	224	0.84 ± 0.55	0.75 [0.49–1.03]		236	1.23 ± 0.93	0.99 [0.65–1.55]		229	1.91 ± 1.64	1.33 [0.79–2.47]	
III	200	0.82 ± 0.49	0.74 [0.51–1.00]		209	1.32 ± 0.93	1.10 [0.74–1.54]		206	1.98 ± 1.66	1.27 [0.89–2.42]	
IV	122	0.86 ± 0.51	0.76 [0.53–1.10]	0.0013^a^	130	1.38 ± 0.99	1.12 [0.72–1.73]	0.0012^a^	124	2.02 ± 1.60	1.49 [0.95–2.35]	0.77
I + II (Ref)	312	0.79 ± 0.51	0.71 [0.46–0.98]		331	1.19 ± 0.89	0.95 [0.63–1.47]		320	1.96 ± 1.75	1.34 [0.79–2.43]	
III + IV	322	0.84 ± 0.50	0.75 [0.52–1.03]	0.3	339	1.34 ± 0.95	1.10 [0.74–1.57]	0.095	330	2.00 ± 1.63	1.36 [0.91–2.39]	0.99
Tumour localisation	*n* = 656				*n* = 693				*n* = 672			
Proximal (C18.0–C18.4) (Ref)	222	0.78 ± 0.47	0.73 [0.46–0.98]		239	1.02 ± 0.67	0.87 [0.62–1.2]		231	1.73 ± 1.59	1.11 [0.79–1.86]	
Distal (C18.5–C19)	272	0.79 ± 0.42	0.71 [0.51–0.98]	0.70	286	1.32 ± 1.07	1.03 [0.65–1.53]	0.0057^a^	277	2.03 ± 1.78	1.37 [0.89–2.42]	0.18
Rectum (C20)	162	0.92 ± 0.66	0.80 [0.54–1.11]	0.023^a^	168	1.46 ± 0.86	1.27 [0.80–1.91]	< 0.0001^a^	164	2.11 ± 1.57	1.50 [0.93–3.05]	0.031^a^
Distal (C18.5–C19) (Ref)	272	0.79 ± 0.42	0.71 [0.51–0.98]		286	1.32 ± 1.07	1.03 [0.65–1.53]		277	2.03 ± 1.78	1.37 [0.89–2.42]	
Rectum (C20)	162	0.92 ± 0.66	0.80 [0.54–1.11]	0.017^a^	168	1.46 ± 0.86	1.27 [0.80–1.91]	0.25	164	2.11 ± 1.57	1.50 [0.93–3.05]	0.65
MSI status	*n* = 588				*n* = 614				*n* = 604			
Stable	523	0.81 ± 0.49	0.73 [0.49–1.00]		553	1.34 ± 0.96	1.12 [0.75–1.62]		544	2.10 ± 1.72	1.44 [0.93–2.60]	
Unstable	57	0.69 ± 0.36	0.67 [0.39–0.91]	0.098	61	0.90 ± 0.52	0.72 [0.61–1.07]	0.0012^a^	60	1.83 ± 1.72	1.39 [0.77–1.99]	0.33
Tumour histology	*n* = 353				*n* = 367				*n* = 358			
Mucinous carcinoma	75	0.79 ± 0.54	0.65 [0.49–0.93]		80	1.16 ± 0.64	1.00 [0.66–1.44]		77	1.90 ± 1.55	1.41 [0.92–2.14]	
Tubular carcinoma	278	0.74 ± 0.52	0.62 [0.41–0.91]	0.28	287	1.78 ± 1.10	1.46 [1.07–2.13]	< 0.0001^a^	281	3.00 ± 1.88	2.31 [1.54–3.98]	<0.0001^a^
Tumour grade	*n* = 629				*n* = 662				*n* = 644			
Low-grade	519	0.81 ± 0.52	0.72 [0.49–1.01]		546	1.29 ± 0.93	1.01 [0.70–1.57]		532	2.02 ± 1.68	1.39 [0.89–2.52]	
High-grade	110	0.88 ± 0.43	0.82 [0.58–1.10]	0.16	116	1.13 ± 0.88	1.0 [0.64–1.32]	0.13	112	1.68 ± 1.71	0.99 [0.72–1.87]	0.051

^a^Numbers of patients may not add up to 100% of available subjects because of RTL measurement failure or due to the lack of clinicopathological data

The study on CRC patients included paired tumour tissues and adjacent non-malignant mucosa from 721 individuals collected during surgical resection. In addition, peripheral blood was available from 164 of those patients, sampled prior to surgery. Further, we also had access to primary tumours, adjacent mucosa, liver metastatic tissues, and paired adjacent liver tissue from 12 individuals from this group of patients.

In order to investigate TL in metastatic liver tissue on a more robust cohort, we also included metastatic and adjacent liver tissues from different 122 CRC patients without primary tumours in the study.

The median age for the group of CRC patients, for whom tissue pairs were available, at the time of diagnosis was 68 years (range 33–96 years); of those, 62.9% were men and 37.1% were women. The patients for whom metastasised tissues were available without primary tumours, had median age of 63 years (range 39–79 years); 66.1% men and 33.9% women.

DNA was extracted from tumour tissues, non-affected adjacent mucosa, blood, metastatic tissues, and liver samples using the DNeasy Blood and Tissue Kit (Qiagen, Courtaboeuf, France). The study was approved by the local ethics committee of each participating hospital. Written informed consent to participate in the study and to approve the use of biological samples for genetic analyses was obtained from all patients, according to the Helsinki declaration.

### Relative telomere length (RTL)

TL was measured as RTL by a Monochrome Multiplex Real-Time qPCR Assay as described previously with some modifications.^12–16^ Syto 9, a single fluorescent dye, was used in real-time qPCR for the collection of a signal from telomere (T; repeat copy number) and albumin (S; human single-copy gene) amplicons, two target sequences greatly differing in copy number. The values of cycle threshold (Ct) for albumin amplicon were collected above the melting temperature of the telomere product. the final TL value was calculated as a ratio between the telomere and albumin product. Measurement of TL from all DNA samples was carried out in triplicate using a MicroAmp Optical 384-Well Reaction Plate (Applied Biosystems, Foster City, CA, USA). A standard twofold serial dilution was prepared based on the known initial concentration of reference DNA (genomic DNA pooled from 30 individuals with 40–55 years age range and without any specific sex ratio^13^). Real-time qPCR experiments were performed on a Viia 7 Real-time PCR System (Applied Biosystems) using two simultaneous programmes to acquire the respective Ct values for telomere sequences and the albumin gene (primer sequences in Supplementary Table 1). The standard curve was used to quantify telomere and albumin genes based on the respective Ct values, and the obtained triplicate values were averaged.^17^ TL was expressed as the ratio between the T/S. Inter-assay and intra-assay variations were determined by duplicating the reference DNA for all dilutions in each assay performed. If T/S ratio exceeded 1, the DNA sample had longer telomeres. On the contrary, if T/S ratio was lower than 1, the DNA sample had shorter telomeres. PCR efficiency for TL measurement varied between 95 and 102%. Inter-plate variation for T and S was 3.5% and 3.4%, respectively. Intra-plate variation for T was 0.42% and 0.28% for S.

### Microsatellite instability (MSI)

MSI status was determined by the molecular testing of five mononucleotide repeat markers (Bethesda consensus panel, BAT 25, BAT 26, NR 21, NR 24, and NR 27) that were run as a pentaplex, using fluorescently labelled primers and standard PCR. Fragment analysis was performed on ABI 3130 (Applied Biosystems). A final comparison between tumour and non-tumour DNA short tandem repetition profiles was analysed with GeneMapper v4.1 software (Applied Biosystems). Tumour specimen was classified as MSI when two or more loci were unstable.

### Statistical analysis

TL measured in the tissues and blood cells of CRC patients was expressed as median and range to characterise individual groups (by means of SAS descriptive statistics, see below).

Statistical analyses were conducted on natural data by using non-parametrical tests. Differences between TL in two distinct tissues were analysed using non-parametrical ANOVA (Wilcoxon signed-rank test). An interquartile range was defined as the distribution of TL values between 25th and 75th percentiles. The relationship between the patient age at diagnosis and TL values was calculated by Pearson correlation coefficient. the TL ratio was expressed as TL in tumour/TL in adjacent mucosa. If the TL ratio exceeded 1, telomeres were longer in tumour tissue than the adjacent mucosa. The curves for overall survival (OS) were derived by the Kaplan–Meier log-rank test. OS was defined as the time from the surgery to the date of death, or the date of the last follow-up. Based on the TL cut-off, all CRC patients were stratified into two groups. Statistical analyses were conducted using SAS Institute Inc. software (Cary, NC, USA). The final results were graphically illustrated using Prism8 GraphPad software (San Diego, CA, USA) and software Statistica (StatSofg, Inc., Tulsa, OK, USA). Statistical significance for all tests was set at *P*-value = 0.05.

## Results

Table 1 summarises TL data in tumour and adjacent mucosa tissues of CRC patients, along with various personal and clinicopathological characteristics. A moderate inverse relationship between TL in adjacent mucosa and age was recorded (*R* = −0.176 and *P* = 0.029).

### TL in tumour tissue, adjacent mucosa, peripheral blood lymphocytes (PBL), liver metastases, and adjacent liver tissues

**Fig. 1:**
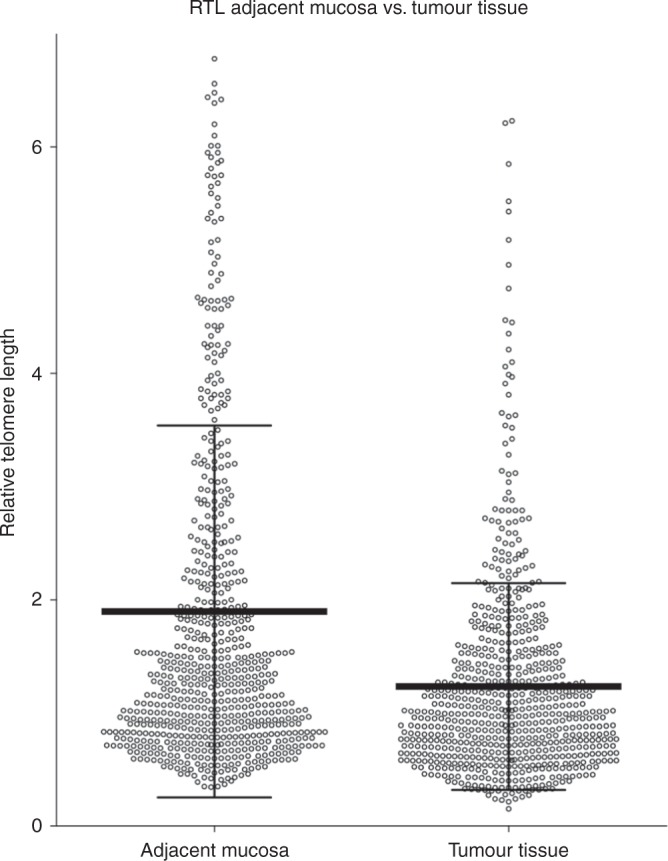
Comparison of RTL in adjacent mucosa and tumour tissue (*P* < 0.0001)

TL in tumour tissues (*n* = 696) was statistically significantly shorter (median [interquartile range]: 0.99 [0.65–1.50]) than in the adjacent mucosa (*n* = 677, 1.29 [0.83–2.26], Wilcoxon test, *P* < 0.0001; Fig. 1). Shorter TL in tumour tissue (i.e. TL ratio < 1) was observed in tumours from 74% patients, while for the remaining 26% of CRC patients, tumours had a longer TL than the adjacent mucosa (based on 661 comparisons, where the data for TL in both tissues were available). We did not find any difference between TL in PBL (*n* = 164, 0.76 [0.56–1.04]) and the corresponding tumour tissues (0.78 [0.58–1.04], *P* = 0.2). Neither there was a correlation between TL in PBL and adjacent mucosa (*R* = 0.026).

TL was statistically significantly higher in 10 (83%) primary tumour tissue samples (1.62 [1.34–1.94]) than in the respective liver metastases (0.71 [0.52–1.01], *P* < 0.0001), which was measured in tumours from only 12 CRC patients.

**Fig. 2:**
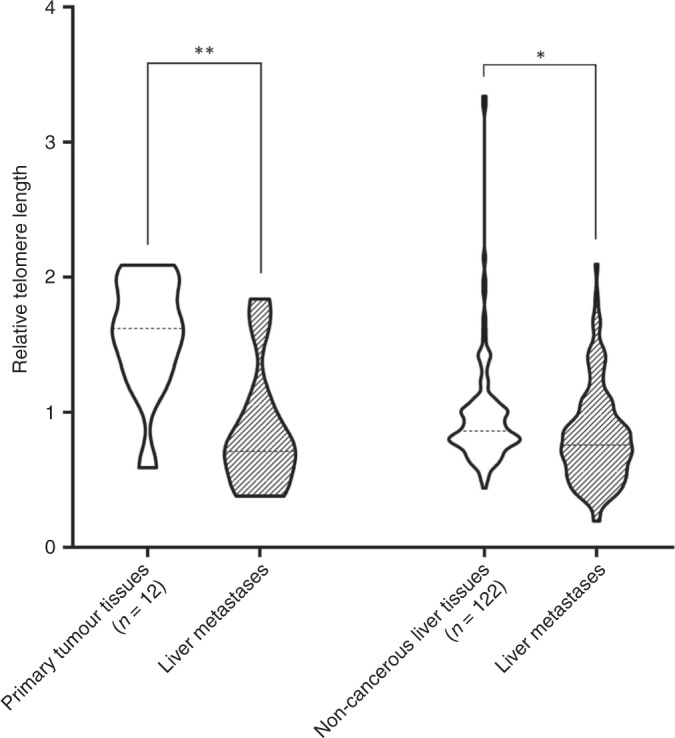
Comparison of RTL in paired tumour and liver metastatic tissues (**P < 0.0001) and in non-cancerous liver tissues and liver metastases (*P = 0.0005)

TL was statistically significantly (*P* = 0.0005) shorter in a group of liver-metastasised tumours (0.76 [0.56–0.98]) than in the adjacent non-cancerous liver tissues (0.86 [0.76–1.02]) from 122 patients (Fig. 2).

### TL vs. tumour localisation

**Fig. 3:**
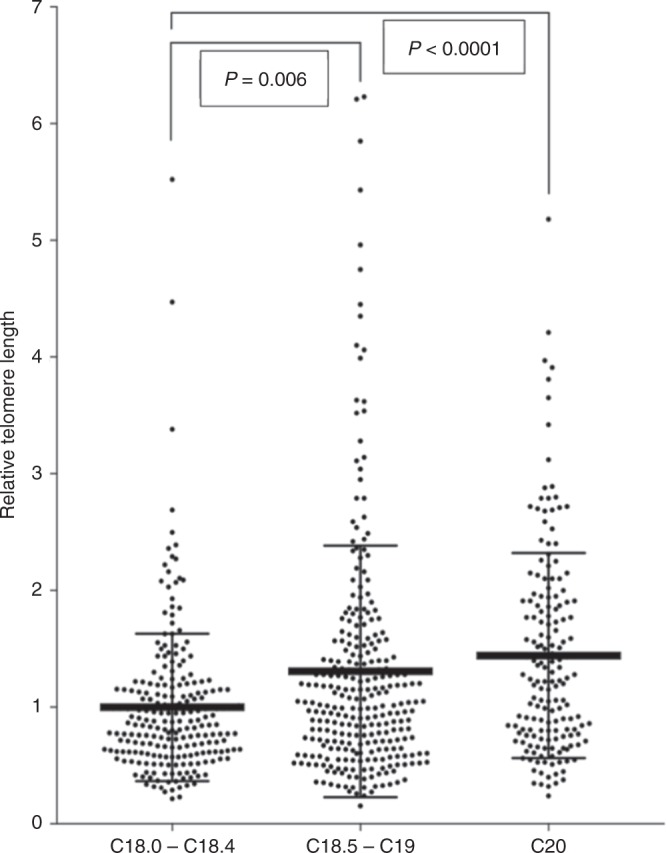
Association between RTL in tumour tissue and tumour site origin; proximal *vs.* distal site (*P* = 0.006), proximal site *vs.* rectum (*P* < 0.0001)

TL in tumour tissues was shorter in the proximal colon ((diagnosis C18.0–C18.4), 0.87 [0.62–1.2]) than in the distal part of the colon (C18.5–C19, 1.03 [0.65–1.53], *P* = 0.006) and rectum (C20, 1.27 [0.8–1.91], *P* < 0.0001; Fig. 3). Adjacent mucosa in CRC patients with proximal tumour origin had the shortest TL (1.11 [0.79–1.86]). However, the TL in the adjacent mucosa of distally located colon tumours (1.37 [0.89–2.42]) was not statistically significantly higher (*P* = 0.18) than the non-malignant mucosa in proximal colon. Interestingly, TL in adjacent mucosa within the proximal tumour origin was statistically significantly shorter than the TL in non-cancerous mucosa attributable to rectal tumours (1.50 [0.93–3.05], *P* = 0.03).

### TL vs. MSI status

Tumour tissues from patients with MSI had statistically significantly shorter TL (*n* = 61, 0.72 [0.60–1.04]) than the microsatellite-stable (MSS) tumours (*n* = 553, 0.99 [0.70–1.30], *P* = 0.009). MSI tumours localised within the proximal part of the colon (*n* = 51, 83.6% from all MSI patients) had statistically significantly shorter TL in tumour tissue (0.72 [0.61–1.07]) than the MSS tumours in proximal colon (1.12 [0.75–1.62], *P* = 0.001). MSS tumours in the proximal colon also had statistically significantly shorter TL than MSS tumours from the rectum (*P* < 0.0001).

### TL vs. tumour node metastasis status

**Fig. 4:**
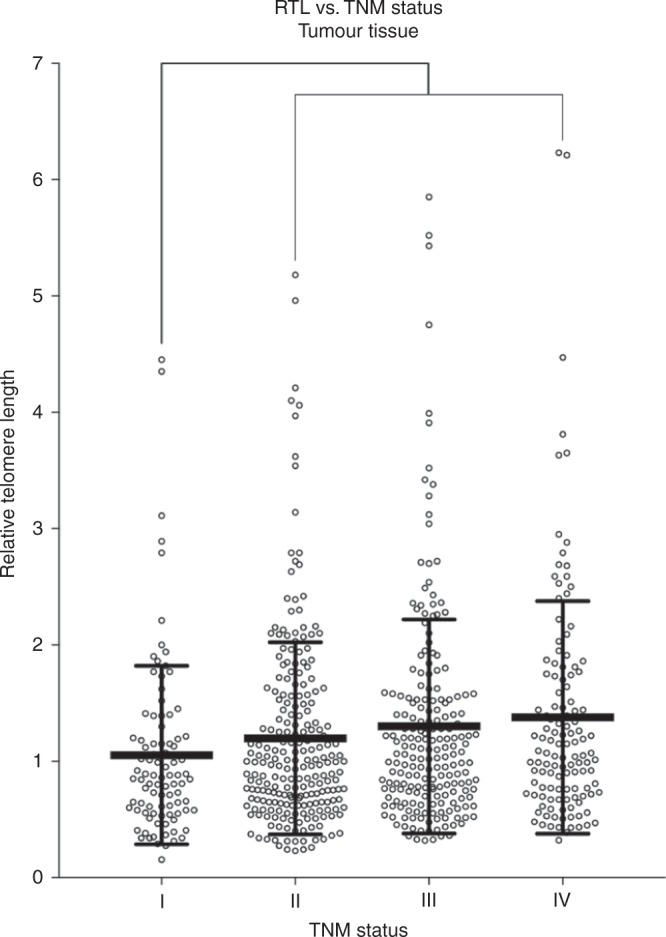
Association between RTL in tumour tissue and TNM status (*P* = 0.001)

Increased TL was observed in tumours with increased TNM stages. A similar trend was not observed in the adjacent mucosa. Patients with TNM stage I had statistically significantly shorter TL in tumour tissue (*P* = 0.001; Fig. 4) than in tumours with TNM stages II + III + IV. The TL ratio in the colon TNM stage I was statistically significantly lower than TNM stages II + III + IV (*P* = 0.001). Any difference in the TL ratio was not observed in the TNM stages of rectal tumours.

### TL vs. tumour histology

TL was statistically significantly shorter in mucinous cancer tissue (*n* = 80, 1.00 [0.66–1.44]) than in tubular cancer tissue (*n* = 287, 1.46 [1.07–2.13], *P* < 0.0001). TL in the adjacent mucosa of patients diagnosed to have mucinous tumours (*n* = 77, 1.41 [0.92–2.14]) was also statistically significantly shorter than the adjacent mucosa of CRC patients with tubular carcinoma (*n* = 281, 2.31 [1.54–3.98], *P* < 0.0001).

### OS vs. TL ratio

**Fig. 5:**
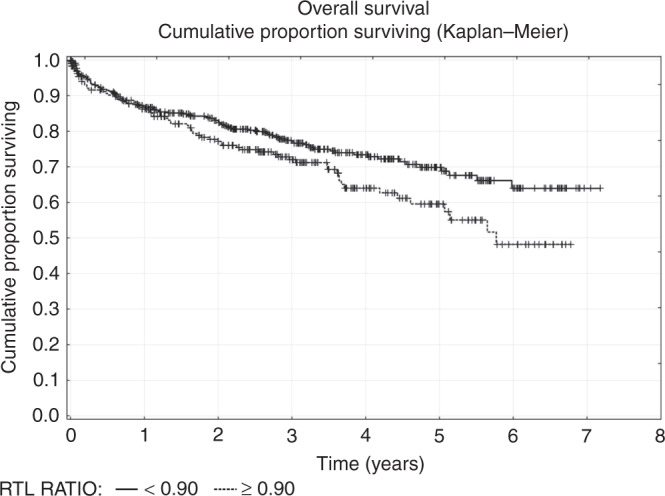
Kaplan–Meier OS curves stratified for the RTL ratio (*P* = 0.022)

Patients with a TL ratio higher than 0.90 had a statistically significantly poorer OS than those patients with lower TL ratios (*P* = 0.02; Fig. 5). We also analysed the possible effect of TL differences on OS following the stratification for MSS and MSI tumour characteristics. We did not observe any significant difference in OS between CRC patients with MSI or MSS tumours (*P* = 0.16).

## Discussion

Our study comprehensively investigated TL in particular bowel segments and in relation to multiple clinicopathological characteristics. This design of the study may conform to the major molecular subtypes of CRC, reflect tumour heterogeneity, and ultimately associate TL with different treatment regimens. Furthermore, our study has addressed TL in metastatic tissues for the first time.

We observed that the telomeres were consistently shorter in a majority of tumour tissues than in adjacent mucosa. The proliferating activity of the rapidly growing tumours underlies this phenomenon as described in some studies carried out in smaller groups of CRC patients.^9,10,18^ However, some studies have also shown contradictory results.^19,20^ Shorter TL in comparison with adjacent mucosa was observed in tumours from 74% patients, while we recorded longer TL in tumours of the remaining 26% of CRC patients. It may be connected with increasing TL in a gradient from the proximal colon to the rectum and with TNM, since we observed statistically significantly shorter TL in the tumours classified as TNM stage I than in tumours with advanced stages of the disease. These differences in TL in tumours and TNM could be due to (a) different molecular/epigenetic subtypes of the tumour, (b) the tumours with longer telomeres may have increased possibility of advancing to higher disease stages than the tumours with short telomeres, and (c) efficient rejuvenation of telomeres in tumour cells.

The existence of two types of CRCs based on TL has been suggested and hypothesised, assuming that TL in healthy tissue might influence telomere maintenance mechanisms in the tumour.^11^ We did observe a gradient increase in TL in the adjacent mucosa from the proximal colon to rectum (data not shown); however, our data presented as TL ratio do indicate an increased TL in distal colon and rectum.

We also demonstrated a prolonged OS in CRC patients with a decreased TL ratio between tumour tissues and the adjacent mucosa. There is a controversy in assessing the prognostic value of TL in CRC. Some studies have identified a longer TL in tumours or higher tumour to non-malignant tissue TL ratios as predictors of poor prognosis,^18,21,22^ others were inconsistent.^10,11^ It is possible that longer telomeres in tumour tissue are associated with their greater proliferation potential and therefore unfavourable prognosis for patients. As reviewed, the ultimate regeneration of a telomerase leading to stochastic levels at critical points rescues cells with short telomeres and high chromosomal instability for infinite proliferation.^2^

The determination of TL in paired primary tumours and metastatic lesions and paired liver samples and metastatic lesions showed excessive telomere erosion in the vast majority of metastases. Since all patients with distant metastases underwent various regimens of chemotherapy, short telomeres could be related to the treatment.^23^

Studies investigating the relationship between MSI status and TL remain rare and inconsistent.^9,24^ Boardman et al.^24^ reported that MSS CIN–rectal cancers have statistically significantly longer telomeres than MSS CIN + rectal samples. We observed shorter telomeres in MSI tumour tissues, occurring predominantly in the proximal part of the colon^25–27^ than in MSS tumours. The results highlight differences in the molecular carcinogenesis between the segments of the colon and rectum.^28^ The assumption that TL in malignant tissue is influenced by the site of tumour origin has been previously addressed by several authors, some of which are contradictory, probably owing to limited sample sizes.^11^

Previous studies^9^^,29^ conducted on 55 and 118 CRC patients, respectively, support our observations of shorter TL in MSI CRC patients than in MSS patients. An explanation for pronounced shorter telomeres in MSI tumours may be the accumulation of unfixed deletions caused by DNA polymerase slippage events during DNA replication, as suggested by Chatterjee and Walker.^30^ Such deletions could result in decreased levels of shelterin subunits at telomeres resulting in a disruption of telomere homoeostasis.^31^ We also observed that MSS tumours in the proximal colon had shorter telomeres than those arising in the rectum, which is in accordance with earlier reports.^9^ Therefore, MSI status and MMR deficiency may only partly explain shorter telomeres in tumours arising at the proximal colon.

Mucinous histology can be counted as another characteristic of MSI tumours. Even though mucinous tumours are predominantly located in the proximal colon, the proportion of patients with mucinous histology in our cohort was not statistically significantly different in relation to other tumour origin sites. However, we found that patients with mucinous tumour histology had statistically significantly shorter TL in the tumour and adjacent mucosa than the patients with tubular carcinoma. Currently, the results in this field are scarce.

Overall, TL was shorter in tumour tissues than in adjacent mucosa, in lower (initial) stages, in the proximal colon and tumours with MSI instability. Furthermore, metastases originating from primary CRC tumours had shorter telomeres than the adjacent non-cancerous liver tissues. Finally, the smaller TL ratio between tumour tissues and adjacent mucosa, conferring to shorter telomeres in tumour, may represent a positive prognostic factor.

The characteristics of TL in relation to the CRC heterogeneity emerge as an urgent predictive issue since the advent of therapeutical concepts based on targeting telomere or telomerase inhibition, in order to overcome resistance, lack of drug sensitivity, toxicity, etc. The understanding of TL in CRC with different clinicopathological features will be an important step.

## Supplementary information

Supplementary Table 1

## Data Availability

The data are available in coded form at the Department of Molecular Biology of Cancer, Institute of Experimental Medicine.

## References

[1] Blackburn EH, Greider CW, Szostak JW (2006). Telomeres and telomerase: the path from maize, Tetrahymena and yeast to human cancer and aging. Nat. Med..

[2] Heidenreich B, Kumar R (2017). TERT promoter mutations in telomere biology. Mutation Res..

[3] Kawanishi S, Oikawa S (2004). Mechanism of telomere shortening by oxidative stress. Ann. N. Y. Acad. Sci..

[4] Savage Sharon A. (2018). Beginning at the ends: telomeres and human disease. F1000Research.

[5] Stewenius Y, Gorunova L, Jonson T, Larsson N, Hoglund M, Mandahl N (2005). Structural and numerical chromosome changes in colon cancer develop through telomere-mediated anaphase bridges, not through mitotic multipolarity. Proc.Natl Acad. Sci. USA..

[6] Grady WM, Markowitz SD (2015). The molecular pathogenesis of colorectal cancer and its potential application to colorectal cancer screening. Dig. Dis. Sci..

[7] Carethers JM, Jung BH (2015). Genetics and genetic biomarkers in sporadic colorectal Cancer. Gastroenterology..

[8] Aaltonen LA, Peltomaki P, Leach FS, Sistonen P, Pylkkanen L, Mecklin JP (1993). Clues to the pathogenesis of familial colorectal cancer. Science.

[9] Rampazzo E, Bertorelle R, Serra L, Terrin L, Candiotto C, Pucciarelli S (2010). Relationship between telomere shortening, genetic instability, and site of tumour origin in colorectal cancers. Br. J. Cancer..

[10] Suraweera N, Mouradov D, Li S, Jorissen RN, Hampson D, Ghosh A (2016). Relative telomere lengths in tumor and normal mucosa are related to disease progression and chromosome instability profiles in colorectal cancer. Oncotarget..

[11] Balc’h EL, Grandin N, Demattei MV, Guyetant S, Tallet A, Pages JC (2017). Measurement of telomere length in colorectal cancers for improved molecular diagnosis. Int. J. Mol. Sci..

[12] Cawthon RM (2009). Telomere length measurement by a novel monochrome multiplex quantitative PCR method. Nucleic Acids Res..

[13] Rachakonda S, Srinivas N, Mahmoudpour SH, Garcia-Casado Z, Requena C, Traves V (2018). Telomere length and survival in primary cutaneous melanoma patients. Sci. Rep..

[14] Kroupa M, Polivkova Z, Rachakonda S, Schneiderova M, Vodenkova S, Buchler T (2018). Bleomycin-induced chromosomal damage and shortening of telomeres in peripheral blood lymphocytes of incident cancer patients. Genes Chromosomes Cancer..

[15] Rachakonda S, Kong H, Srinivas N, Garcia-Casado Z, Requena C, Fallah M (2018). Telomere length, telomerase reverse transcriptase promoter mutations, and melanoma risk. Genes Chromosomes Cancer..

[16] Srinivas N, Rachakonda S, Hielscher T, Calderazzo S, Rudnai P, Gurzau E (2019). Telomere length, arsenic exposure and risk of basal cell carcinoma of skin. Carcinogenesis..

[17] Bustin SA, Benes V, Garson JA, Hellemans J, Huggett J, Kubista M (2009). The MIQE guidelines: minimum information for publication of quantitative real-time PCR experiments. Clin. Chem..

[18] Gertler R, Rosenberg R, Stricker D, Friederichs J, Hoos A, Werner M (2004). Telomere length and human telomerase reverse transcriptase expression as markers for progression and prognosis of colorectal carcinoma. J. Clin. Oncol..

[19] Katayama S, Shiota G, Oshimura M, Kawasaki H (1999). Clinical usefulness of telomerase activity and telomere length in the preoperative diagnosis of gastric and colorectal cancer. J. Cancer Res. Clin. Oncol..

[20] O’Sullivan J, Risques RA, Mandelson MT, Chen L, Brentnall TA, Bronner MP (2006). Telomere length in the colon declines with age: a relation to colorectal cancer? Cancer epidemiology, biomarkers & prevention: a publication of the American Association for Cancer Research, cosponsored by the American Society of Preventive. Oncology..

[21] Garcia-Aranda C, de Juan C, Diaz-Lopez A, Sanchez-Pernaute A, Torres AJ, Diaz-Rubio E (2006). Correlations of telomere length, telomerase activity, and telomeric-repeat binding factor 1 expression in colorectal carcinoma. Cancer..

[22] Valls C, Pinol C, Rene JM, Buenestado J, Vinas J (2011). Telomere length is a prognostic factor for overall survival in colorectal cancer. Colorectal Dis..

[23] Benitez-Buelga C, Sanchez-Barroso L, Gallardo M, Apellániz-Ruiz M, Inglada-Pérez L, Yanowski K (2015). Impact of chemotherapy on telomere length in sporadic and familial breast cancer patients. Breast Cancer Res.Treat..

[24] Boardman LA, Johnson RA, Viker KB, Hafner KA, Jenkins RB, Riegert-Johnson DL (2013). correlation of chromosomal instability, telomere length and telomere maintenance in microsatellite stable rectal cancer: a molecular subclass of rectal cancer. Plos One..

[25] Walther A, Houlston R, Tomlinson I (2008). Association between chromosomal instability and prognosis in colorectal cancer: a meta-analysis. Gut..

[26] Yamauchi M, Lochhead P, Morikawa T, Huttenhower C, Chan AT, Giovannucci E (2012). Colorectal cancer: a tale of two sides or a continuum?. Gut..

[27] Missiaglia E, Jacobs B, D’Ario G, Di Narzo AF, Soneson C, Budinska E (2014). Distal and proximal colon cancers differ in terms of molecular, pathological, and clinical features. Ann.Oncol..

[28] Paschke S, Jafarov S, Staib L, Kreuser ED, Maulbecker-Armstrong C, Roitman M (2018). Are colon and rectal cancer two different tumor entities? A proposal to abandon the term colorectal cancer. Int. J. Mol.Sci..

[29] Takagi S, Kinouchi Y, Hiwatashi N, Nagashima F, Chida M, Takahashi S (2000). Relationship between microsatellite instability and telomere shortening in colorectal cancer. Dis. Colon Rectum..

[30] Chatterjee N, Walker GC (2017). Mechanisms of DNA damage, repair, and mutagenesis. Environ. Mol. Mutagenesis..

[31] Loayza D, De Lange T (2003). POT1 as a terminal transducer of TRF1 telomere length control. Nature..

