# Supplementary material for: Relationship of telomere length in colorectal cancer patients with cancer phenotype and patient prognosis
Source: Br J Cancer. 2019 Jul 17;121(4):344–50. doi: 10.1038/s41416-019-0525-3 (PMC6738117; doi:10.1038/s41416-019-0525-3)
Supplement: Supplementary file 1 — Supplementary Table 1 [file 41416_2019_525_MOESM1_ESM.docx]

| **Telg** | 5′ ACACTAAGGTTTGGGTTTGGGTTTGGGTTTGGGTTAGTGT 3′ |
| --- | --- |
| **Telc** | 5′ TGTTAGGTATCCCTATCCCTATCCCTATCCCTATCCCTAACA 3′ |
| **Albugcr2** | 5′ CGGCGGCGGGCGGCGCGGGCTGGGCGGCCATGCTTTTCAGCTCTGCAAGTC 3′ |
| **Albdgcr2** | 5′ GCCCGGCCCGCCGCGCCCGTCCCGCCGAGCATTAAGCTCTTTGGCAACGTAGGTTTC 3′ |

**Supplementary Table 1:** Primers for RTL measurement
